# Tablet-Based Patient-Centered Decision Support for Minor Head Injury in the Emergency Department: Pilot Study

**DOI:** 10.2196/mhealth.8732

**Published:** 2017-09-28

**Authors:** Navdeep Singh, Erik Hess, George Guo, Adam Sharp, Brian Huang, Maggie Breslin, Edward Melnick

**Affiliations:** ^1^ Medical College of Georgia AU/UGA Medical Partnership Athens, GA United States; ^2^ Department of Emergency Medicine Yale School of Medicine New Haven, CT United States; ^3^ Department of Emergency Medicine Mayo Clinic Rochester, MN United States; ^4^ Department of Research and Evaluation Kaiser Permanente Southern California Pasadena, CA United States; ^5^ Department of Emergency Medicine Los Angeles Medical Center Kaiser Permanente Southern California Los Angeles, CA United States; ^6^ School of Visual Arts New York, NY United States

**Keywords:** clinical decision support, decision aids, head injury, minor, medical informatics, spiral computed tomography, health services overuse, patient-centered outcomes research

## Abstract

**Background:**

The Concussion or Brain Bleed app is a clinician- and patient-facing electronic tool to guide decisions about head computed tomography (CT) use in patients presenting to the emergency department (ED) with minor head injury. This app integrates a patient decision aid and clinical decision support (using the Canadian CT Head Rule, CCHR) at the bedside on a tablet computer to promote conversations around individualized risk and patients’ specific concerns within the ED context.

**Objective:**

The objective of this study was to describe the use of the Concussion or Brain Bleed app in a high-volume ED and to establish preliminary efficacy estimates on patient experience, clinician experience, health care utilization, and patient safety. These data will guide the planning of a larger multicenter trial testing the effectiveness of the Concussion or Brain Bleed app.

**Methods:**

We conducted a prospective pilot study of adult (age 18-65 years) patients presenting to the ED after minor head injury who were identified by participating clinicians as low risk by the CCHR. The primary outcome was patient knowledge regarding the injury, risks, and CT use. Secondary outcomes included patient satisfaction, decisional conflict, trust in physician, clinician acceptability, system usability, Net Promoter scores, head CT rate, and patient safety at 7 days.

**Results:**

We enrolled 41 patients cared for by 29 different clinicians. Patient knowledge increased after the use of the app (questions correct out of 9: pre-encounter, 3.3 vs postencounter, 4.7; mean difference 1.4, 95% CI 0.8-2.0). Patients reported a mean of 11.7 (SD 13.5) on the Decisional Conflict Scale and 92.5 (SD 12.0) in the Trust in Physician Scale (both scales range from 0 to 100). Most patients were satisfied with the app’s clarity of information (35, 85%), helpfulness of information (36, 88%), and amount of information (36, 88%). In the 41 encounters, most clinicians thought the information was somewhat or extremely helpful to the patient (35, 85%), would want to use something similar for other decisions (27, 66%), and would recommend the app to other providers (28, 68%). Clinicians reported a mean system usability score of 85.1 (SD 15; scale from 0 to 100 with 85 in the “excellent” acceptability range). The total Net Promoter Score was 36.6 (on a scale from –100 to 100). A total of 7 (17%) patients received a head CT in the ED. No patients had a missed clinically important brain injury at 7 days.

**Conclusions:**

An app to help patients assess the utility of CT imaging after head injury in the ED increased patient knowledge. Nearly all clinicians reported the app to be helpful to patients. The high degree of patient satisfaction, clinician acceptability, and system usability support rigorous testing of the app in a larger multicenter trial.

## Introduction

One-third of patients with minor head injury receive head computed tomography (CT) that may not be clinically indicated [1-6]. These potentially avoidable CTs do not change management. However, they do increase health care costs, exposure to ionizing radiation, and length of stay in the emergency department (ED) [7]. The American Board of Internal Medicine and the American College of Emergency Physicians’ Choosing Wisely initiative have recognized this and recommend avoiding unnecessary head CTs in patients with minor head injuries as the top national priority for addressing CT overuse in emergency care [1]. The Canadian CT Head Rule (CCHR) is a clinical decision rule that was developed using a rigorous, evidence-based derivation and validation process to identify minor head injury in patients at risk for clinically important structural brain injuries and the need for neurosurgical intervention. A total of 7 history and physical criteria are used as indications for CT based on their association with these risks. This rule was designed to safely reduce head CT use in patients with minor head injury. It has been validated to be 100% sensitive in detecting patients needing neurosurgical intervention. Additionally, the CCHR outperforms other decision rules with the highest specificity in its class [8-11].

Implementing the CCHR with traditional computerized clinical decision support (CDS) has had a modest effect (5%-8%) on decreasing CT use in these patients [12,13]. Since one-third of CTs in minor head injury patients are potentially avoidable and traditional CDS has had limited effect on reducing these scans, it has been hypothesized that nonclinical factors (such as fear of litigation, physician personality, fear of missed diagnoses, financial incentives, paucity of information, and patient expectations) also contribute to CT overuse in these patients [14,15]. Qualitative research on this topic revealed that physician-based empathic factors such as establishing trust and engaging patients by identifying and addressing their concerns are essential to reduce CT overuse [15,16].

**Figure 1:**
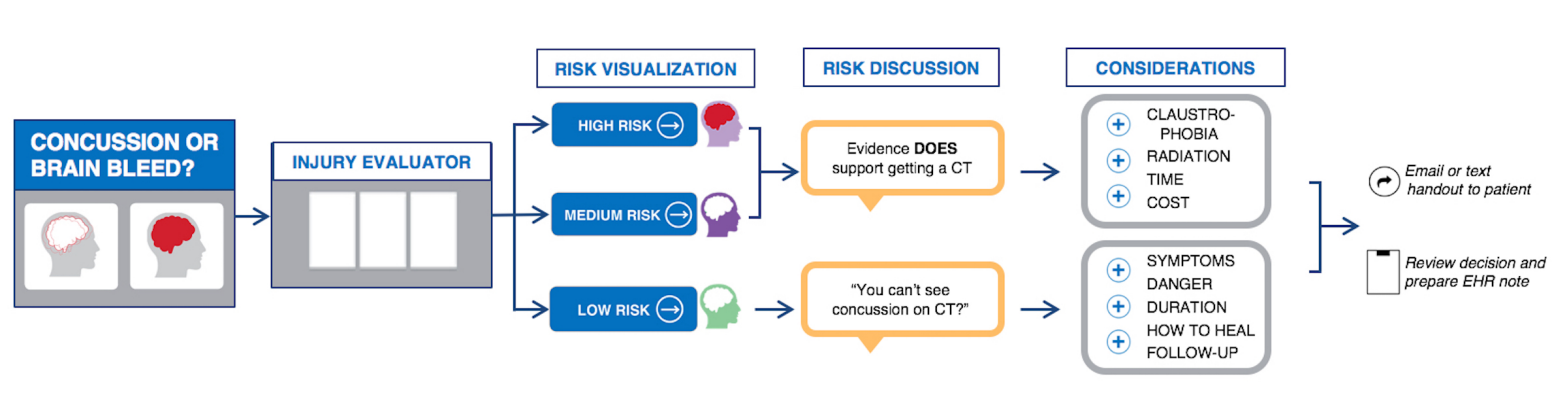
Conceptualization of the workflow and potential pathways for the Concussion or Brain Bleed app. CT: computed tomography; EHR: electronic health record.

We previously developed a clinician- and patient-facing electronic tool to guide decisions about CT use in ED patients with minor head injury, called Concussion or Brain Bleed [17,18]. This app integrates a patient decision aid and CDS (using the CCHR) at the bedside on a tablet computer to promote conversations around individualized risk and patients’ specific concerns within the ED’s clinical constraints [19,20]. Although intended primarily for use in low-risk patients, the app includes pathways for moderate- and high-risk patients as well. Figure 1 presents the conceptual workflow of the app: (1) welcome screen, (2) injury evaluator (CDS portion), (3) risk visualization, (4) risk discussion with conversation prompts such as “You can’t see concussion on CT?,” (5) considerations, and (6) integration back to traditional workflow (the paper handout given to patients after using the intervention is publicly available [21]).

**Figure 2:**
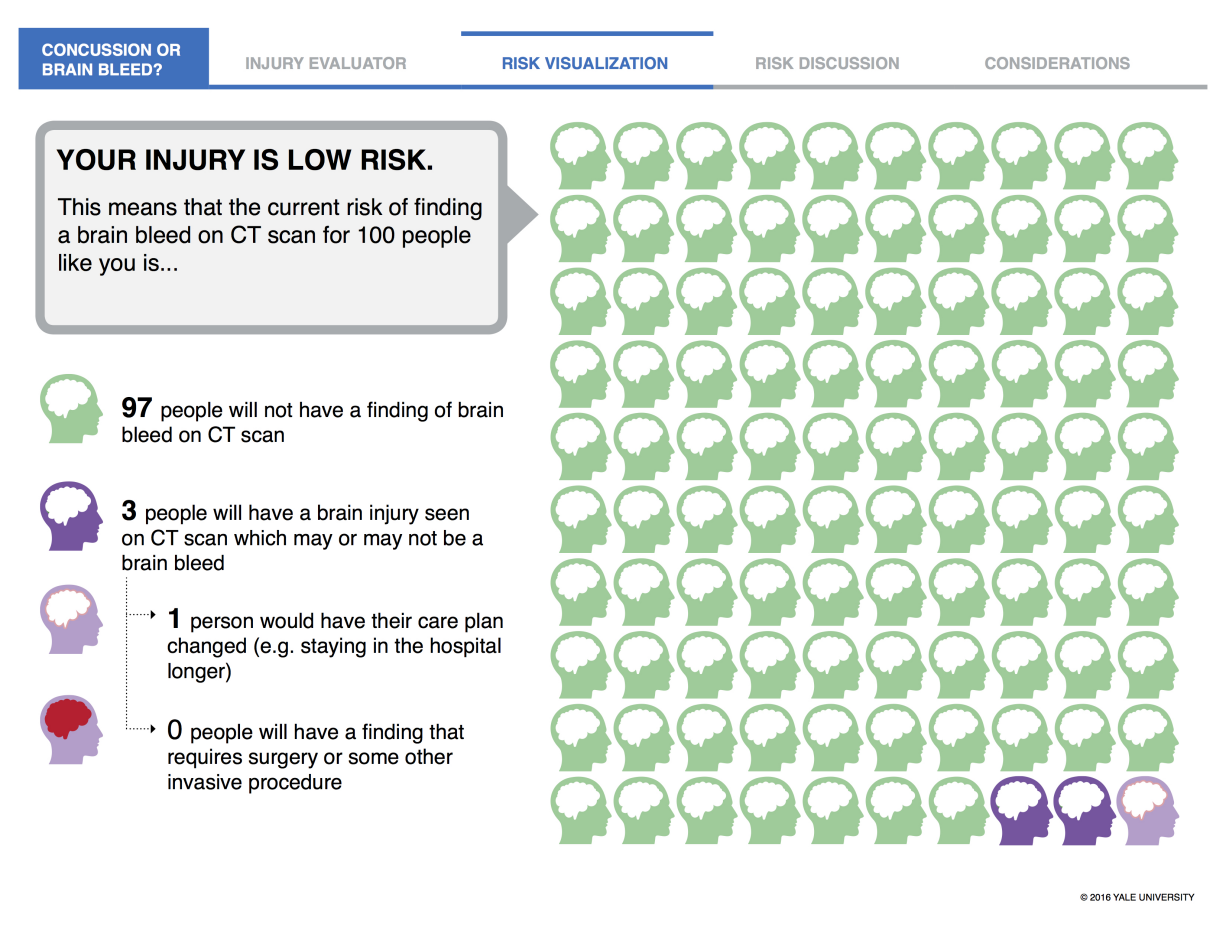
Risk visualization screen shot for low-risk patients from the Concussion or Brain Bleed app. CT: computed tomography.

Figure 2 presents the risk visualization screen for the low-risk pathway. The long-term implementation goal for this patient-centered decision support tool is to safely and effectively reduce CT use for patients with minor head injury while simultaneously improving the patient experience. In the trial presented here, our objective was to describe the use of the Concussion or Brain Bleed app in a high-volume ED and to establish preliminary efficacy estimates on patient experience, clinician experience, health care utilization, and patient safety.

## Methods

### Study Design, Setting, and Population

**Figure 3:**
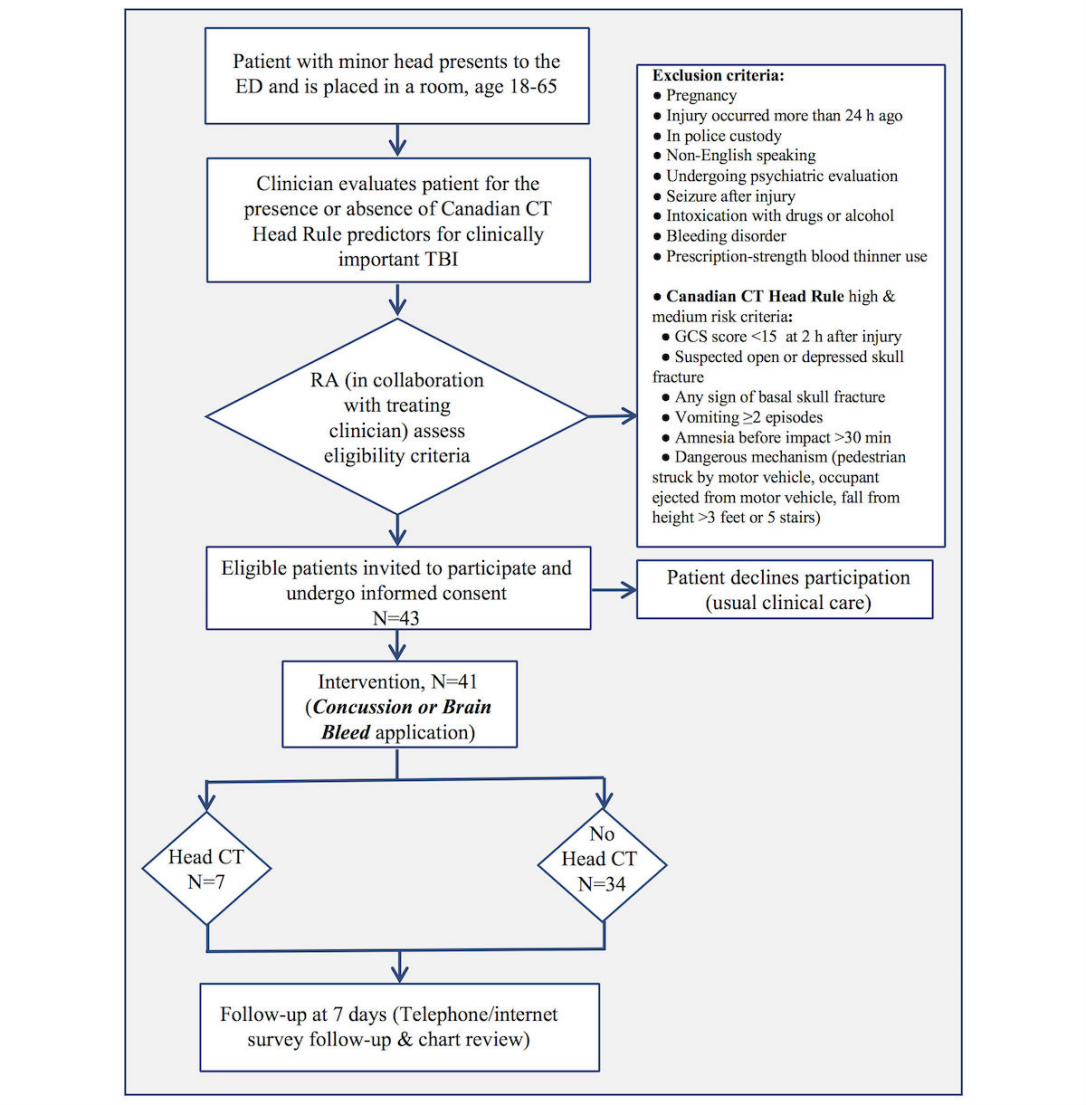
Flow diagram for patient identification and enrollment in the flow of patient care with number of patients identified, enrolled, and receiving computed tomography (CT) in the emergency department (ED). GCS: Glasgow Coma Scale; RA: research assistant; TBI: traumatic brain injury.

We performed a prospective pilot study with a convenience sample of 41 ED patients with minor head injury. Patients were enrolled over a 6-week period (May 23 to July 3, 2017). Patients and clinicians who were eligible and willing to participate used the Concussion or Brain Bleed app and completed a survey to determine the app’s baseline efficacy on patient experience, clinician experience, health care utilization, and patient safety. Participants were patients and clinicians recruited from an urban, academic level I trauma center ED with 103,000 patient-visits per year. Eligible patients were adults (age 18-65 years) presenting to the ED who had experienced blunt head injury within the last 24 hours who were determined to be at low risk by the CCHR (see Figure 3) and were being considered for head CT imaging by the treating clinician. Patients who were pregnant, non-English speaking, in police custody, undergoing psychiatric evaluation, or found to have drug or alcohol intoxication were excluded. Eligible clinicians were attending physicians, fellows, residents, and midlevel providers caring for eligible patients. We recruited clinicians from the 48 attending physician faculty, 58 resident physicians, and 47 midlevel providers. The study protocol was approved by our institutional review board (IRB), the Yale Human Investigation Committee.

### Participant Identification, Recruitment, and Enrollment

A research assistant (RA; NS) reviewed an electronic patient tracking board at regular intervals to identify potentially eligible patients based on a chief complaint potentially consistent with head trauma. Next, the RA worked with the clinician assigned to the patient’s care team to determine whether the patient met inclusion criteria (either before or after the initial clinical evaluation). Next, the clinician and patient were informed about the study and asked if they would be willing to participate. The participating clinician and patient provided verbal consent as specified by the IRB-approved protocol. We collected all data using the Web-based survey tool Qualtrics Survey Tool (Qualtrics, LLC), on Yale’s electronic patient health information-approved and certified, licensed online platform. Clinicians were compensated for participation in the study with a US $10 gift card to a coffee shop.

### Training

The RA gave participating clinicians a brief (<2 minute) tutorial of the Concussion or Brain Bleed app prior to using it the first time. This individualized, just-in-time training provided an opportunity to highlight each section of the app and to demonstrate its navigation. The clinician was given an opportunity to ask any additional questions or to repeat sections of the training as needed until they felt comfortable with its use. Although RAs were available at the point of care to assist with any technical issues or difficulty navigating the app on an as-needed basis, they refrained from interfering with the actual use of the app to observe an accurate representation of its use in routine care.

### Patient and Clinician Characteristics

We collected patient demographics by self-report at the time of enrollment, including age, sex, race, ethnicity, highest level of education, insurance status, and household income. Patient literacy and numeracy were assessed immediately before use of the app using the validated Subjective Literacy Scale and Subjective Numeracy Scale [22-24]. The Subjective Literacy Scale comprises 3 items, each rated on a 5-point Likert scale and summed into a total score of 3-15. The Subjective Numeracy Scale consists of 8 items that assesses comfort in working with numbers, each rated on a 6-point Likert scale with an overall score ranging from 6 to 48.

We collected clinician characteristics by self-report following the clinician’s first use of the tool. Clinician characteristics that were collected included demographics, years practicing emergency medicine, medical degree or role, and details on personal technology use.

### Outcome Measures

Outcome selection was informed by a similar study performed by Hess et al [25] using a paper-based, shared decision-making aid in a pediatric population to compare the decision aid’s effectiveness with usual care on (1) parent knowledge regarding their child’s risk, diagnostic options, and risks associated with CT, (2) parent engagement in the decision-making process, (3) degree of conflict parents experience related to feeling uninformed, (4) patient and clinician satisfaction, (5) rate of clinically important traumatic brain injury at 7 days, (6) proportion of patients in whom a CT scan was obtained, and (7) 7-day health care utilization [25]. That study selected outcomes based on input from key stakeholders, including patient representatives, practicing clinicians, researchers (including shared decision-making experts), and health policy decision makers. Patient knowledge was selected as the primary outcome for that study based on input from patient representatives. For our study reported here, we selected patient knowledge as the primary outcome and other secondary outcomes based on this precedent from the pediatric shared decision-making study, including the Decisional Conflict Scale, the Trust in Physician Scale, similar satisfaction, health care utilization, and patient safety outcomes [25].

### Patient Outcomes

#### Patient Knowledge

We assessed patient knowledge using a pre- and postvisit survey administered immediately before and after the clinical encounter (Multimedia Appendix 1) [25]. In the survey, 9 questions assessed patients’ knowledge regarding concussion, their individual risk of structural brain injury, the available diagnostic options, the risks related to radiation exposure associated with a head CT scan, the potential for a CT scan to identify incidental abnormalities that may require further investigation, and reasons to return to the ED for reevaluation should their symptoms worsen after ED discharge. We calculated the percentage of knowledge questions answered correctly to determine the mean difference between knowledge scores before and after use of the intervention.

#### Decisional Conflict

We measured the patient’s degree of conflict with the decision of whether to get a CT scan using the validated Decisional Conflict Scale [25-28]. The 16 items on this scale are scored on a scale 0 to 4; the items are summed, divided by 16, and then multiplied by 25. The scale ranges from 0 to 100, where higher scores reflect patient uncertainty about the choice.

#### Trust in the Physician

We measured patients’ trust in their clinician using the validated Trust in Physician Scale [25,28-30]. This scale has 10 items, which are scored on a scale of 1 to 5; the items are summed, divided by 10, and then multiplied by 100. The scale ranges from 0 to 100, where higher values reflect higher levels of trust in their clinician.

#### Patient Satisfaction

We measured patients’ satisfaction with the way information was shared during the encounter by asking 5 questions using a 7-point Likert scale. For the analysis, we classified satisfaction into satisfied/very satisfied versus other responses.

### Clinician Outcomes

#### Clinician Satisfaction

We assessed clinician satisfaction immediately after the patient encounter via a questionnaire regarding the helpfulness of the app and the clinician’s satisfaction with the way information was shared on a 7-point Likert scale. For the analysis, we classified satisfaction into satisfied/very satisfied versus other responses.

#### System Usability Scale

The System Usability Scale consists of a 10-item questionnaire on a 5-point Likert scale that gives a reliable assessment of usability [31]. The 10 items of the System Usability Scale are scored on a scale of 0 to 4, with each even-numbered question reverse coded. The items are summed and then multiplied by 2.5. Scores range from 0 to 100, where higher scores indicate higher usability.

#### Net Promoter Score

The Net Promoter Score has been employed across industries to measure how willing a user is to recommend a product or service to others [32]. A higher score on this scale ranging from –100 to 100 can indicate a greater growth rate of the corresponding product or service. We determined the score by first asking the clinician user on a scale from 0 to 10 (0=not likely at all, 10=extremely likely) “How likely are you to recommend the Concussion or Brain Bleed application to a colleague?” If a clinician answered 9 or 10, we categorized them as a “promoter”—someone who would enthusiastically recommend the app to others. If a clinician answered 6 or lower, we considered them to be a “detractor”—someone who would potentially give a negative review to others. The Net Promoter Score is calculated by subtracting the percentage of promoters from the percentage of detractors. We calculated a total Net Promoter Score factoring in all encounters in which the app was used, as well as a first-time user Net Promoter Score and a second-time user Net Promoter Score.

#### Fidelity Score

We assessed the fidelity with which the intervention was delivered and used as intended using a fidelity checklist of 8 intended actions (see Multimedia Appendix 2). The fidelity checklist has been used in the absence of the intervention to check for contamination in the usual-care arm of a trial [25].

### Health Care Utilization and Patient Safety

CT scans were obtained at the ED clinicians’ discretion and interpreted by site faculty radiologists. The main health care utilization outcome was the proportion of patients for whom head CT was obtained in the ED. We also collected data at the time of the ED visit (and confirmed by chart review) on (1) whether the patient was admitted to the hospital, (2) acute findings on CT if obtained, and (3) whether the clinician reported that they would have made the same decision regarding CT imaging without using the app. The RA contacted enrolled patients by telephone or email starting at 7 days after the index ED visit to ensure no outcomes were missed. The 7-day follow-up was based on timing of delayed clinical deterioration and our previous work [8,25].

### Analysis Plan

Results are reported using descriptive statistics and stratified by patient and clinician outcomes. The unit of analysis was the ED encounter. We defined change in patient knowledge as the mean difference of questions answered correctly pre- and postencounter. We performed analysis in Microsoft Excel (version 2016; Microsoft Corporation) on data exported from the Qualtrics Survey Tool. We made every effort to minimize the occurrence of missing data. We attempted to verify (or ascertain, if missing) items self-reported by patients at the 7-day follow-up by medical record review. We report rates of missing data as well as known reasons for missing data. We conducted secondary exploratory analyses of variables predictive of the odds of CT imaging, patient knowledge, and trust in physician using univariate logistic and linear regression with SAS (version 9.3; SAS Institute).

## Results

### Patient and Clinician Characteristics

We enrolled 41 of 43 identified patients (see Figure 1; recruitment rate 95%) in the 6-week study period with a mean age of 34.9 years (range 18-59; see Table 1). The majority of patients were female (26, 63%), were not of Hispanic or Latino origin (31, 76%), and identified high school or general educational diploma or less as their highest level of education (24, 59%). The mean patient subjective literacy score was 12.4 (SD 2.8), and mean subjective numeracy score was 30.4 (SD 8.5).

Of 33 eligible clinicians, 29 (recruitment rate 88%) caring for eligible patients agreed to participate. The mean clinician age was 34 years (range 24-51; see Table 2). The majority of clinicians were female (15, 52%), not of Hispanic or Latino origin (36, 90%), white (20, 69%) and physicians (MDs) (16, 55%). There were 11 (38%) clinicians with a Physician Assistant degree and 2 (7%) with an Advanced Practice Registered Nurse (nurse practitioner) degree. The mean (range) years of experience practicing emergency medicine (including residency) was 5.8 (0-24). All clinicians owned a personal smartphone (29, 100%) and most owned a personal tablet computer (21, 72%). The majority of clinicians (24, 83%) also indicated they spent over 30 hours a week on a computer, tablet, or smartphone.

### Patient Experience

Mean (SD) knowledge assessment scores increased from 3.3 (1.9) out of 9 pre-encounter to 4.7 (2.1) postencounter (Multimedia Appendix 1), with mean difference of 1.4 (95% CI 0.8-2.0, see Table 3). The mean (SD) patient decisional conflict score was 11.7 (13.5), and the mean (SD) trust in physician score was 92.5 (12). Both scales are from 0 to 100. Patient satisfaction scores showed that a majority of patients were satisfied with the clarity of information (35, 85%), helpfulness of the information (36, 88%), and amount of information (36, 88%). The majority of patients also said that they would recommend the app to others (36, 88%) and would want to use something similar for other clinical decisions (26, 63%).

The mean (SD) fidelity score was 6.7 (1.8; see Table 5) out of the 8 intended actions that the app aimed to elicit. Clinicians most consistently described the different risk levels portrayed on the risk visualization pictograph (95%). Clinicians least frequently elicited the patient or caregiver’s concerns (61%).

### Health Care Utilization and Patient Safety

In the 41 encounters in which the app was used, 7 patients (17%; see Table 6) received a head CT in the ED. Since these patients were at low risk, all 7 CTs scans were not recommended based on the CCHR criteria. Of the 7 CTs, the 3 most frequently cited reasons for obtaining CT were referring physician request (5/7, 71%), mechanism of injury (3/7, 43%), and headache (3/7, 43%). In 100% of cases in which the app was used, clinicians reported they would make the same decision without the app. No patients were admitted to the hospital (0, 0%). Follow-up data were collected via phone call from 34 patients (83%), email from 4 patients (10%), and chart review for the remaining 3 patients (7%). At 7-day follow-up, 4 patients (10%) had returned to an ED, 14 patients (34%) had visited a physician's office or clinic, 1 patient (2%) did both, and 22 patients (54%) did neither. Further testing or procedures were obtained for 5 patients (12%) within 7 days following the encounter, and 2 patients (5%) underwent neuroimaging within 7 days. No patient had acute findings on CT in the ED or on follow-up imaging (0%).

### Secondary Analyses

On secondary analyses of variables predictive of the odds of CT imaging, fidelity with the concerns portion of the intervention (odds ratio 0.19, 95% CI 0.03-1.15, *P*=.07), not having low literacy (odds ratio 0.23, 95% CI 0.04-1.26, *P*=.09), and system usability score above average (odds ratio 0.24, 95% CI 0.03-1.83, *P*=.17) trended toward significance but these results were not statistically significant. Patient knowledge and trust in the physician yielded no statistically significant results. Variables that trended toward significance for change in patient knowledge from pre- to postencounter in univariate analysis were white patient race (variable 1.14, 95% CI –0.14 to 2.41, *P*=.08), fidelity with the concerns portion of the intervention (variable –0.83, 95% CI –2.13 to 0.47, *P*=.21), not having low literacy and not having low numeracy (both with variable 0.64, 95% CI –0.66 to 1.94, *P*=.33) but these results were not statistically significant. Variables that trended toward significance for trust in physician on univariate analysis were white patient race (variable 5.89, 95% CI –1.29 to 13.08, *P*=.11), fidelity with the concerns portion of the intervention (variable 3.59, 95% CI –3.74 to 10.91, *P*=.34), and not having low literacy (variable 3.83, 95% CI –4.02 to 11.68, *P*=.34) but these results were not statistically significant.

**Table 1:** Patient characteristics.

Characteristics		Data
Participants recruited, n		43
Participants enrolled, n (%)		41 (95)
Age (years), mean (range)		34.9 (18-59)
Female, n (%)		26 (63)
**Race, n (%)**		
	Black or African American	15 (37)
	White	17 (42)
	Asian	1 (2)
	American Indian or Alaska Native	1 (2.4)
	Other	9 (22)
**Ethnicity, n (%)**		
	Hispanic or Latino origin	10 (24)
	Not of Hispanic or Latino origin	31 (76)
**Education, n (%)**		
	Some high school or less	4 (10)
	High school graduate	20 (49)
	Some college	12 (29)
	College graduate or more	5 (12)
**Insurance, n (%)**		
	Private/HMO^a^	21 (51)
	Medicaid only	17 (42)
	Medicare only	0 (0)
	Medicare + Medicaid	1 (2)
	Uninsured	2 (5)
**Annual household income (US $), n (%)**		
	<20,000	8 (20)
	20,000-29,999	6 (15)
	30,000-39,999	6 (15)
	40,000-59,999	4 (10)
	60,000-79,999	7 (17)
	80,000-99,999	5 (12)
	≥100,000 or more	5 (12)
Subjective Literacy Scale score, mean (SD)		12.4 (2.8)
Subjective Numeracy Scale score, mean (SD)		30.4 (8.5)

^a^HMO: health maintenance organization.

**Table 2:** Clinician characteristics.

Characteristics			Data
Participants recruited, n			33
Participants enrolled, n (%)			29 (88)
Age (years), mean (range)			34 (24-51)
Female, n (%)			15 (52)
**Race, n (%)**			
	White		20 (69)
	Asian		8 (28)
	Other		2 (7)
**Ethnicity, n (%)**			
	Hispanic or Latino origin		3 (10)
	Not of Hispanic or Latino origin		36 (90)
**Medical degree, n (%)**			
	Advanced Practice Registered Nurse		2 (7)
	Physician Assistant		11 (38)
	Physician (MD)		16 (55)
Experience (years), mean (range)			5.8 (0-24)
**Technology use, n (%)**			
	**Time (hours) spent on a computer, tablet, or smartphone per week**		
		<10	0 (0)
		10-20	2 (7)
		20-30	3 (10)
		30-40	8 (28)
		>40	16 (55)
	**Preferred method of contact**		
		Call on landline	0 (0)
		Call on mobile phone	9 (31)
		Email	0 (0)
		Text	20 (69)
		Other	0 (0)
	**Mobile technology use**		
		Personal tablet computer	21 (72)
		Personal smartphone	29 (100)

**Table 3:** Patient experience outcomes and results.

Outcome			Data
**Patient knowledge**			
	**Knowledge (no. questions correct out of 9), mean (SD)**		
		Pre-encounter	3.3 (1.9)
		Postencounter	4.7 (2.1)
		Mean difference (95% CI)	1.4 (0.8-2.0)
**Decisional conflict and trust**			
	Decisional Conflict Scale (scale of 0-100), mean (SD)		11.7 (13.5)
	Trust in Physician Scale (scale of 0-100), mean (SD)		92.5 (12.0)
**Patient satisfaction, n (%)**			
	**Amount of information**		
		Too little (1-2)	0 (0)
		Just right (3-5)	36 (88)
		Too much (6-7)	5 (12)
	**Clarity of information**		
		Satisfied (5-7)	35 (85)
		Unsatisfied (1-4)	6 (15)
	**Helpfulness of information**		
		Satisfied (5-7)	36 (88)
		Unsatisfied (1-4)	5 (12)
	**Would recommend to others**		
		Yes (1-3)	36 (88)
		Not sure/no (4-7)	5 (12)
	**Would want to use for other decisions**		
		Yes (1-3)	26 (63)
		Not sure/no (4-7)	15 (37)

**Table 4:** Clinician experience outcomes and results.

Outcome			Data
**System usability and net promoter scores**			
	System Usability Scale score (scale of 0-100), mean (SD)		85.1 (15.0)
	Total Net Promoter Score (scale of –100 to 100)		36.6
	First-time user Net Promoter Score		31.0
	Second-time user Net Promoter Score		50.0
**Clinician acceptability, n (%)**			
	**Helpfulness of the information**		
		Not helpful at all (1-2)	1 (2)
		Somewhat helpful (3-5)	16 (39)
		Extremely helpful (6-7)	24 (59)
	**Would want to use for other decisions**		
		Yes (1-2)	27 (66)
		Not sure (3-5)	13 (32)
		No (6-7)	1 (2)
	**Would recommend to others**		
		Yes (1-2)	28 (68)
		Not sure (3-5)	13 (32)
		No (6-7)	0 (0)

**Table 5:** Fidelity score and compliance with delivery of the intervention as intended.

Fidelity of Use Assessment Question		n (%)
Did the clinician describe how the severity of the injury was evaluated using the Canadian CT^a^Head Rule?		37 (90)
Did the clinician describe the risk as a natural frequency (eg, “of 100 people like you, 6 will...”)?		37 (90)
Did the clinician describe the different risk levels portrayed on the risk visualization pictograph?		39 (95)
Did the clinician explain the difference between concussion and brain bleed?		31 (76)
Did the clinician explain what kinds of injuries can and cannot be seen on a CT scan?		33 (81)
Did the clinician elicit the patient and/or caregiver’s concerns?		25 (61)
Did the clinician discuss the patient and/or caregiver’s specific concerns?		35 (85)
**(Follow-up Discussion)**		
	(If no CT performed) Did the clinician discuss what to expect after leaving the ED?	36 (88)
	(If CT performed) Did the clinician discuss issues to consider before getting a CT scan?	0 (0)
Total score out of 8 possible, mean (SD)		6.7 (1.6)

^a^CT: computed tomography.

**Table 6:** Health care utilization and patient safety results.

Outcome	n (%)
Head CT^a^obtained in the ED^b^	7 (17)
Clinician would make same decision without the app	41 (100)
Admitted to the hospital	0 (0)
Acute findings on CT in ED	0 (0)
ED return visit within 7 days	4 (10)
Physician office or clinic visit within 7 days	14 (34)
Both ED return visit and physician office or clinic visit within 7 days	1 (2)
Neither ED return visit nor physician office or clinic visit within 7 days	22 (54)
Neuroimaging within 7 days	2 (5)
Acute findings on neuroimaging within 7 days	0 (0)

^a^CT: computed tomography.

^b^ED: emergency department.

## Discussion

In patients with low-risk minor head injury who were being considered for CT head imaging in the ED, use of the Concussion or Brain Bleed app in this prospective interventional pilot study resulted in increased patient knowledge and was associated with a low rate of CT use, high trust in the physician, low patient decisional conflict, high clinician Net Promoter Score, and high system usability score without any adverse events in patients. We found the app to be acceptable to both patients and clinicians.

### Comparison With Other Studies

Our trial’s setup was similar to those of other ED shared decision-making trials for adult patients with chest pain and pediatric patients with head injury [25,28]. The high trust in physician and low decisional conflict scores reported here establish baseline efficacy of the Concussion or Brain Bleed app. These scores are consistent with those of previous ED trials of paper-based decision aids for adult ED patients with chest pain (trust in physician: mean 89.5, SD 13.4 versus this study, 92.5, SD 12.0; decisional conflict: mean 43.5, SD 11.3 versus this study, 11.7, SD 13.5) and parents of pediatric ED patients with head injury (results to be reported soon) [25,28]. Although the results have not yet been formally reported, our population had similar but slightly lower literacy and numeracy than the trial studying parents of pediatric ED patients with head injury described in the Outcome Measures subsection above [25].

Traditional implementation strategies lead to increased CT use in minor head injury [33]. On the other hand, traditional CDS has had only a modest effect (5%-8%) on decreasing the rate of CT overuse (35%) in these patients [2,5]. The overuse rate in our study of 17% cuts this rate in half. Based on our previous qualitative work, we hypothesize that this additional decrease was due to the intervention’s ability to engage patients and address nonclinical factors (eg, identifying and addressing patient concerns and increasing physician trust). However, the number of patients enrolled in this study was limited and was a convenience sample [15].

The intervention’s System Usability Scale and Net Promoter scores were also high. To put them in context, a system usability score of 85.1 has been correlated with the adjective rating of “excellent” or a grade of A+ [34,35]. Amazon.com is a frequently used website that has been found to have a similar system usability score [36]. Furthermore, the Net Promoter Score of 36.6 indicates a greater rate of users who were promoters than detractors of the product and, therefore, suggests the product’s growth potential [32].

### Meaning of the Study

Overuse of CT in minor head injury is complex and multifactorial, including both clinical and nonclinical contributing factors [14,15]. Traditional implementation strategies such as CDS can address clinical factors such as a lack of awareness of the evidence [37]. However, these strategies have had limited success for this decision, likely due to nonclinical factors such as patients’ concerns with their condition and care [12-15]. Findings of this study suggest that patients can be educated and engaged in the ED setting in decisions about CT imaging for low-risk minor head injury using a health information technology interface that supports the clinician-patient relationship (rather than getting in its way) [17,38,39]. Specifically, if these findings are confirmed in a larger effectiveness trial, it would imply that successful adoption of the Concussion or Brain Bleed app could help address nonclinical factors that contribute to overuse of CT in minor head injury that are not addressed with traditional implementation strategies and traditional CDS.

### Strengths and Weaknesses of the Study

Unlike traditional implementation efforts, this intervention systematically aims to use technology at the bedside to engage, educate, and reassure patients. This pilot study took place at a single site, so the results may not be generalizable to other EDs. Similarly, unique infrastructure already in place in our ED (but not part of the intervention) could have contributed to the app’s success. This study was conducted by 1 RA who was responsible for enrollment, clinician training, and data collection. An RA provides internal consistency but could be prone to bias based on the RA’s level of performance. Enrollment primarily occurred in the evenings, which is similar to our previous findings on enrollment for head injury patients in the ED [40]. The patients enrolled were representative of the patient population seen in our ED, which serves an urban, underserved population with low literacy and numeracy.

This pilot study has shown that it is feasible to use an integrated decision aid with CDS on a tablet computer at the bedside in the ED to engage, educate, and reassure low-risk minor head injury patients about CT and concussion. This finding is promising but, without a control arm, a conclusion cannot be drawn regarding the intervention’s efficacy in reducing potentially avoidable CT scans. Although only 2 patients and 4 clinicians declined to participate, enrollment of a convenience sample may also have introduced a self-selection bias of clinicians and patients who were more amenable to this type of approach. For example, the clinicians were relatively young and tech savvy (average age 34 years and 69% using text messaging as their preferred method of personal communication). Since diffusion of innovations benefits from early adoption by a population that is likely to be receptive to change and technology, we believe this is a necessary first step to adoption [41].

One of the top priorities of the Concussion or Brain Bleed app is to have the clinician identify and address the patient’s specific concerns. Therefore, we were troubled to note that fidelity with eliciting concerns was the lowest fidelity score of the 8 intended actions that the intervention aimed to elicit (Table 5). To address this, we revised the Risk Discussion screen as discussed in Multimedia Appendix 3.

Based on the secondary analyses, fidelity with identifying and addressing patients’ concerns trended to significance for being predictive of CT imaging rate (odds ratio 0.19, *P*=.07), change in patient knowledge (variable –0.83, *P*=.21), and trust in physician (variable 5.89, *P*=.11) but these results were not statistically significant. These findings are consistent with our qualitative research that identifying and addressing patients’ concerns influences overuse of CT in low-risk minor head injury patients [15]. The findings of the secondary analysis of fidelity with the intervention were consistent with verbal feedback we received from users that it was difficult to *both* educate and address patient concerns due to time constraints. This study was not powered to detect which variables were predictive of outcomes. However, these estimates give us a sense of the direction of association that may exist. The results reported here will help to determine the sample size of future effectiveness research comparing this intervention versus usual care.

### Unanswered Questions and Future Research

In this pilot study, research staff were available to coordinate use of the Concussion and Brain Bleed app in appropriate patients. Given the competing demands in the ED context, in the absence of research staff there would be multiple barriers to its use, adoption, and integration into routine ED care. Although clinicians reported in every use of the intervention that the app did not affect their clinical decision whether to obtain CT imaging, we maintain that the Concussion or Brain Bleed app has the potential to safely reduce CT imaging in low-risk minor head injury patients. Future research should focus on assessing and optimizing the context for implementation of the Concussion or Brain Bleed app into routine ED care. Identifying barriers and facilitators for how best to embed this complex innovation as part of routine care could optimize its reach, effectiveness, adoption, implementation, and maintenance in routine care [42,43]. For example, a qualitative analysis could explore the reasons that some physicians approved of the tool but would not recommend it to others. Once these factors are identified and optimized, our plan to compare the effectiveness of the app versus usual care could more fully determine its effects on patient experience, clinician experience, health care utilization, and patient safety. If the app is effective, our next goal would be to scale the intervention for dissemination and implementation to outside sites. At the time of this publication, the Concussion or Brain Bleed app is also being adapted for use in Canada with plans to study it there in a comparative effectiveness trial as well. Another category of unanswered questions to explore further would be the concept of patient-centered decision support—for example, which clinical decisions are appropriate for patient decision aids versus traditional CDS versus patient-centered integrated solutions like the one presented here.

### Conclusion

An app to help patients assess the utility of CT imaging after head injury in the ED increased patient knowledge, was associated with a low rate of CT overuse, and was reported to be “extremely helpful” to patients. The high degree of patient satisfaction and clinician acceptability, and high system usability scores are evidence to support the need for rigorous testing of the app in future research that could optimize its implementation into routine ED care and measure its effectiveness compared with usual care.

## Appendix

Multimedia Appendix 1 Patient Knowledge Assessment Questionnaire.

Multimedia Appendix 2 Fidelity checklist.

Multimedia Appendix 3 Revisions to Risk Discussion screen based on pilot user feedback.

## Abbreviations

CCHR: Canadian CT Head Rule
CDS: clinical decision support
CT: computed tomography
ED: emergency department
IRB: institutional review board
RA: research assistant
