# Supplementary figures and images for: Tablet-Based Patient-Centered Decision Support for Minor Head Injury in the Emergency Department: Pilot Study
Source: JMIR Mhealth Uhealth. 2017 Sep 28;5(9):e144. doi: 10.2196/mhealth.8732 (PMC5639208; doi:10.2196/mhealth.8732)

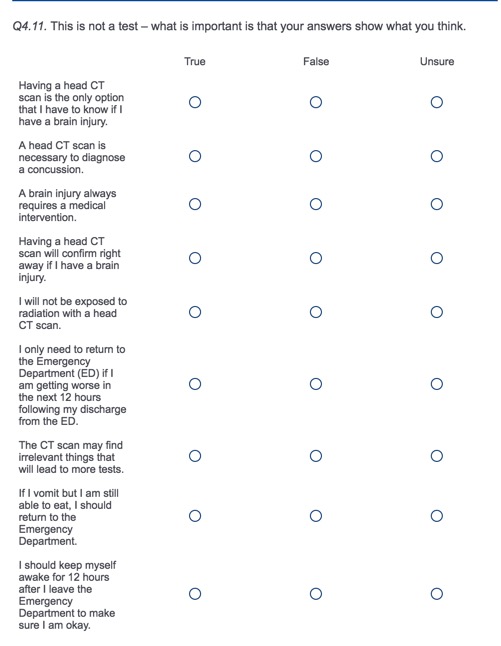

Supplement: Multimedia Appendix 1 [file mhealth_v5i9e144_app1.jpg]
