# Supplementary material for: Tablet-Based Patient-Centered Decision Support for Minor Head Injury in the Emergency Department: Pilot Study
Source: JMIR Mhealth Uhealth. 2017 Sep 28;5(9):e144. doi: 10.2196/mhealth.8732 (PMC5639208; doi:10.2196/mhealth.8732)
Supplement: Multimedia Appendix 2 [file mhealth_v5i9e144_app2.pdf]

Fidelity Checklist

Did the clinician describe how the severity of the injury was evaluated using the Canadian CT Head Rule?

Did the clinician describe the risk as a natural frequency (e.g. “of 100 people like you, 6 will...”)?

Did the clinician describe the different risk levels portrayed on the risk visualization pictograph?

Did the clinician explain the difference between concussion and brain bleed?

Did the clinician explain what kinds of injuries can and cannot be seen on a CT scan?

Did the clinician elicit the patient and/or caregiver’s concerns?

Did the clinician discuss the patient and/or caregiver’s specific concerns?

(Follow-up Discussion)
(If no CT performed) Did the clinician discuss what to expect after leaving the ED?
(If CT performed) Did the clinician discuss issues to consider before getting a CT scan?
