# Supplementary material for: Tablet-Based Patient-Centered Decision Support for Minor Head Injury in the Emergency Department: Pilot Study
Source: JMIR Mhealth Uhealth. 2017 Sep 28;5(9):e144. doi: 10.2196/mhealth.8732 (PMC5639208; doi:10.2196/mhealth.8732)

Risk Discussion Screen used in pilot study:

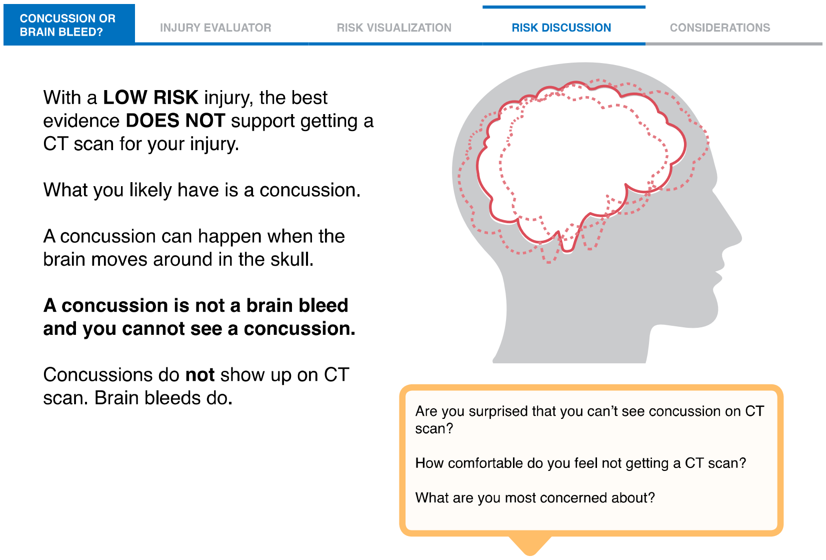

w

We reviewed the Risk Discussion screen with 10 clinician users and gathered their feedback on how to improve it to better elicit patient concerns. This feedback along with literacy accessibility software (Readable.io) were used to make the revisions that are depicted below. These changes were shared with 5 users who all agreed that they were an improvement. Following this revision, the tool was used with 4 more patients (with 3 new clinicians and 1 clinician who had previously used the tool). Clinician training for these subsequent patients placed more careful emphasis on the goal of addressing patient concerns. Clinician elicited concerns in all 4 of these patients.

Revised Risk Discussion screen based on pilot user feedback:

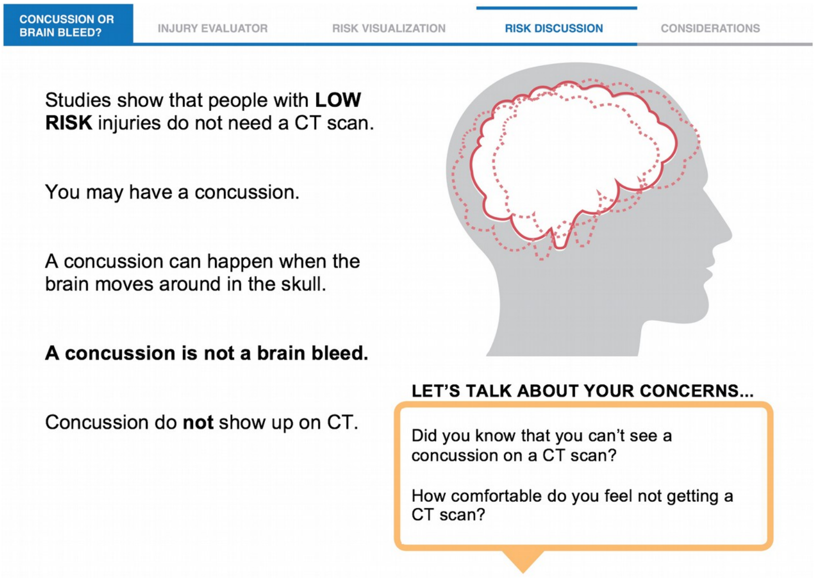

Supplement: Multimedia Appendix 3 [file mhealth_v5i9e144_app3.pdf]
